# Light-induced dynamic shaping and self-division of multipodal polyelectrolyte-surfactant microarchitectures via azobenzene photomechanics

**DOI:** 10.1038/srep41327

**Published:** 2017-01-23

**Authors:** Nicolas Martin, Kamendra P. Sharma, Robert L. Harniman, Robert M. Richardson, Ricky J. Hutchings, Dominic Alibhai, Mei Li, Stephen Mann

**Affiliations:** 1 Centre for Organized Matter Chemistry, School of Chemistry, University of Bristol, Bristol BS8 1TS, UK; 2 Department of Chemistry, Indian Institute of Technology Bombay, Mumbai, 400076, India; 3 School of Physics, H. H. Wills Physics Laboratory, University of Bristol, Tyndall Avenue, Bristol BS8 1TL, UK; 4 Wolfson Bioimaging Facility, Faculty of Biomedical Sciences, University of Bristol, University Walk, Bristol BS8 1TD, UK

## Abstract

Light-induced shape transformations represent a fundamental step towards the emergence of adaptive materials exhibiting photomechanical behaviours. Although a range of covalent azobenzene-based photoactive materials has been demonstrated, the use of dynamic photoisomerization in mesostructured soft solids involving non-covalent co-assembly has received little attention. Here we prepare discrete micrometre-sized hydrated particles of a hexagonally ordered polyelectrolyte-surfactant mesophase based on the electrostatically induced co-assembly of poly(sodium acrylate) (PAA) and *trans-*azobenzene trimethylammonium bromide (*trans-*azoTAB), and demonstrate unusual non-equilibrium substrate-mediated shape transformations to complex multipodal microarchitectures under continuous blue light. The microparticles spontaneously sequester molecular dyes, functional enzymes and oligonucleotides, and undergo self-division when transformed to the *cis* state under UV irradiation. Our results illustrate that weak bonding interactions in polyelectrolyte-azobenzene surfactant mesophases can be exploited for photo-induced long-range molecular motion, and highlight how dynamic shape transformations and autonomous division can be activated by spatially confining azobenzene photomechanics in condensed microparticulate materials.

Achieving stimuli-dependent control over shape transformations in artificial assemblies represents a fundamental step for the design and construction of adaptive materials exhibiting emergent behaviours^1^,^2^. Within this context, light represents an attractive external energy source for the remote control of morphological transitions in synthetic materials at high spatiotemporal resolution. Photochromic materials based on azobenzene derivatives are particularly attractive for the implementation of photo-mechanical responses owing to their robust and reversible *trans-cis* light-induced isomerisation that affects both the molecular volume and orientation of the transition dipole moment^3^,^4^,^5^,^6^,^7^. Shape deformations involving bending, twisting, expansion-contraction, swelling, melting or oscillations have been observed in crystalline molecular assemblies^8^,^9^,^10^,^11^,^12^, microgel particles^13^, azobenzene-containing polymer films^14^,^15^,^16^,^17^ and liquid crystal azobenzene-containing elastomeric polymers^18^,^19^,^20^,^21^,^22^,^23^. Repeatable deformations are usually achieved in these materials by the sequential use of ultraviolet (UV) and blue light irradiations to induce alternative *trans*-*cis* followed by reverse *cis*-*trans* isomerisation of the azobenzene moieties. Alternating the excitation frequency results in photo-switching between two equilibrium states. In contrast, attempts to design artificial systems responding to continuous light excitation under non-equilibrium conditions have only recently been achieved. For example: directional crawling of azobenzene crystals on a glass surface has been achieved in response to irradiation with spatially oriented UV and blue light sources via localized melting and crystallization at the rear and front edges of the crystals, respectively^10^; a biomimetic peristaltic-like motion has been implemented in a liquid crystalline elastomer microswimmer via photo-isomerisation-induced local contraction-expansion of the soft material under structured dynamic light fields^23^; and surface relief gratings have been spontaneously produced in azobenzene-containing polymer thin films subjected to polarized and structured light sources^16^,^24^,^25^,^26^.

Non-equilibrium behaviour has also been recently achieved without the requirement of spatially organized light stimuli by employing repeated photo-isomerisations under continuous irradiation in spatially confined condensed materials to achieve photostationary states in which Brownian diffusion no longer drives the system to equilibrium. For instance, dual-wavelength excitation has been used to simultaneously generate co-localized *cis* and *trans* isomers in crosslinked liquid crystal polymer networks^27^,^28^ that result in the amplification of deformations. Significantly, similar non-equilibrium effects can be observed under continuous blue light illumination due to the overlapping absorption spectra of the *trans* and *cis* isomers in bi-substituted azobenzene derivatives^24^. Under these conditions, the individual azobenzene molecules are stochastically oscillating between *trans* and *cis* isomers to produce a time-averaged population distribution that represents a platform for molecular motion (bending/unbending) and collective behaviour maintained by the continuous energy input. Switching off the blue light immediately stops the oscillation such that molecules in the *cis* conformation thermally relax exponentially to the *trans* state typically with a mean life time of 25 h in the solution state, although this process can be accelerated in closely packed assemblies^29^.

Activation to the *trans/cis* photostationary state has recently been applied to the design of dissipative supramolecular motors^30^, and induction of autonomous or chaotic oscillations in thin crystalline assemblies^12^ or liquid crystalline polymer films^28^, respectively. Long-distance mass transport is usually inhibited in these systems because of immobilization of the azobenzene molecules into crystalline arrays or via covalent attachment to crosslinked polymer chains^31^, which in turn limit the extent of light-induced shape transformations. In contrast, the bottom-up assembly of photoactive materials based on non-covalent bonding between linear polymers and mesogens^29^,^32^,^33^ could be advantageous for photo-induced long-range molecular motion due to the weaker bonding interactions.

In this paper, we develop a new type of photomechanical mesostructured material in which non-covalent interactions between a polyelectrolyte (poly(sodium acrylate); PAA) and a surfactant (*trans-*azobenzene trimethylammonium bromide; *trans-*azoTAB) facilitate long-range molecular motion under non-polarized continuous blue light irradiation (Fig. 1a). Specifically, we exploit the electrostatic- and hydrophobic-driven cooperative self-assembly in water of cationic *trans-*azoTAB and polyanionic PAA to prepare discrete photoresponsive micrometre-sized hydrated particles of a hexagonally ordered *trans-*azoTAB:PAA mesophase. We demonstrate that the soft microparticles undergo an unusual non-equilibrium substrate-mediated shape transformation to complex multipodal microarchitectures when activated into a spatially confined *trans/cis* photo-stationary state by continuous blue light (450 < λ < 490 nm). Moreover, the hydrated nature of the *trans-*azoTAB:PAA mesostructure facilitates uptake of functional biomolecules and organic dyes, which can then be subsequently transferred into the filamentous extensions of the multipodal *trans/cis-*azoTAB:PAA micro-architecture by exposure to blue light. Finally, we show that individual *trans-*azoTAB:PAA microparticles rapidly transform into single hemispherical particles of isotropically ordered *cis-*azoTAB:PAA when exposed to UV irradiation, whilst UV irradiation of the *trans/cis-*azoTAB:PAA multipodal structures results in spatially controlled division of the filaments into clusters of multiple all-*cis* particles. Taken together, our results demonstrate a facile route to the bottom-up co-assembly of photomechanical polyelectrolyte-surfactant mesostructured particles that are capable of non-equilibrium transformation into complex micro-architectures and autonomous division when adsorbed at water/solid interfaces.

## Results

### Preparation and characterization of *trans*-azoTAB:PAA microparticles

**Figure 1:**
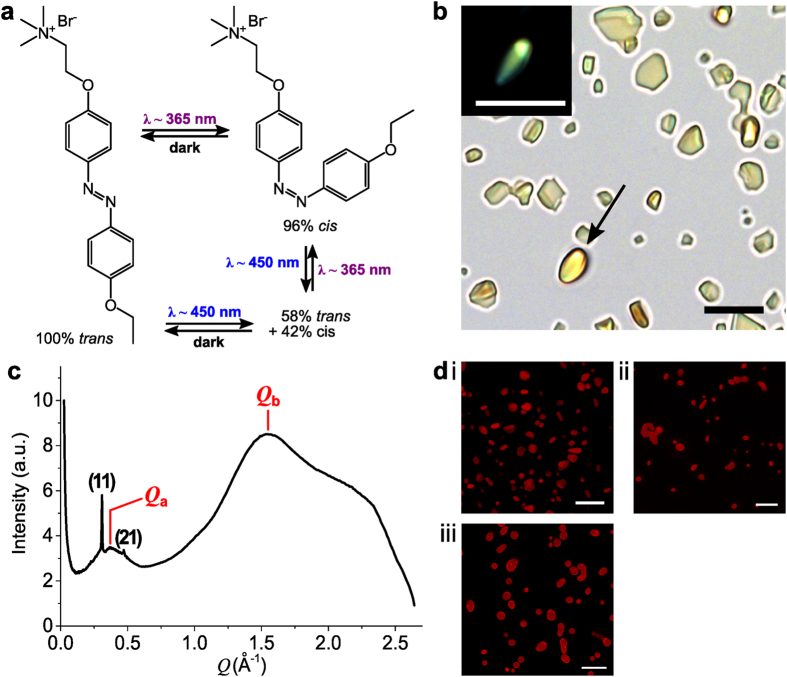
(**a**) Chemical structure of azoTAB and wavelength-dependent photoinduced isomerisation events. (**b**) Optical microscopy image of hydrated *trans*-azoTAB:PAA microparticles mounted on a PEG-functionalized glass slide; inset shows particle highlighted by the arrow and viewed under polarized light; scale bars = 10 μm. (**c**) SAXS profile of hydrated *trans*-azoTAB:PAA showing low angle Bragg reflections corresponding to a hexagonally packed mesostructure of surfactant micelles interspaced with polymer chains and water molecules. Absence of the (10) peak is attributed to the hexagonal axis being parallel to the mica substrate. (**d**) Confocal fluorescence microscopy images of hydrated *trans*-azoTAB:PAA microparticles with sequestered (i) Nile Red (1 μM), (ii) HRP-RITC (50 nM) or (iii) Cy5-ssDNA (1 μM), scale bars = 10 μm.

Addition of PAA (M_w_ = 5.1 kDa) to a clear aqueous solution of *trans-*azoTAB (Fig. 1a) at a concentration (5 mM) below the critical micelle concentration (12.6 mM)^34^ and azoTAB:PAA charge ratio of 2:1 produced a turbid yellow suspension of the polyelectrolyte-surfactant complex, *trans-*azoTAB:PAA (Supplementary Fig. 1). Zeta potential measurements on the aqueous dispersions gave values close to 0 mV. Optical microscopy images showed discrete birefringent particles with noncircular/angular shapes (Fig. 1b) with a circularity of 0.62 ± 0.14 (i.e. ≪1) and average size of 2.6 ± 1.8 μm. Centrifugation of the sample and analysis of the supernatant indicated that the microparticles had a charge ratio of 1:1 with excess aqueous azoTAB remaining in the solution. The hydrated particles were soft solids with a water content of 47 ± 8 wt% (*ca*. 20 water molecules per ion pair of *trans-*azoTAB:PAA), and Young’s modulus of 380 ± 140 kPa (air-dried value = 13.7 ± 0.7 MPa; atomic force microscopy (AFM) nano-mechanical measurements). No melting point or thermal transition was detected by differential scanning calorimetry (DSC) below 90 °C, and the microparticles remained unchanged morphologically when heated up to 80 °C in water. Raman microscopy confirmed the presence of azobenzene stretching modes in the particles (Supplementary Fig. 2). Formation of *trans-*azoTAB:PAA in water was inhibited at high salt concentrations or in the presence of high levels of the azobenzene host molecule hydroxypropyl β-cyclodextrin^35^ (Supplementary Fig. 3), indicating that electrostatic and short-range inter-azobenzene hydrophobic interactions were responsible for self-assembly of the polyelectrolyte-surfactant complex.

Small angle X-ray scattering (SAXS) profiles from bulk samples of hydrated *trans*-azoTAB:PAA prepared by sedimentation of the microparticles onto mica substrates showed two low angle Bragg reflections at *Q* = 0.309 Å^−1^ and 0.472 Å^−1^ that corresponded to the (11) and (21) reflections of a two-dimensional (2D) hexagonal mesostructure with a unit cell parameter of 4.1 nm that was approximately twice the length of the azoTAB molecule^36^ (Fig. 1c). This was consistent with a hexagonally packed array of cylindrical *trans-*azoTAB micelles that were decorated with PAA chains and water molecules at the surface of the trimethylammonium headgroups^37^ (Supplementary Fig. 4). The minimum domain size of the hexagonal mesophase was determined from the width of the Bragg peaks and estimated to be at least 100 nm. 2D SAXS patterns showed continuous rings consistent with an array of randomly oriented domains. The absence of the hexagonal (10) reflection suggested that the *trans-*azoTAB micelles were preferentially oriented parallel to the mica surface; i.e. perpendicular to the incident X-ray beam-light. Wide angle X-ray scattering data from the same samples showed two broad peaks at *Q*_a_ = 0.372 Å^−1^ and *Q*_b_ = 1.537 Å^−1^, along with a shoulder at ~2 Å^−1^ (Fig. 1c), indicating that the surfactant molecules were disordered at the molecular length scale. The *Q*_a_ and *Q*_b_ peaks were assigned to the azoTAB molecular length (~1.7 nm)^33^ and intermolecular distance between adjacent surfactants (~0.4 nm), respectively. The shoulder peak at Q ~ 2 Å^−1^ (d ~ 0.3 nm) was in agreement with reported π-π stacking distances for *trans-*azobenzene groups^38^, suggesting that charge screening of the trimethylammonium headgroups by carboxylate groups of PAA facilitated hydrophobic packing of the azoTAB molecules into the hexagonal mesostructure.

A wide range of molecules including water-soluble and water-insoluble organic dyes (Nile red (neutral), methylene blue (cationic), sulforhodamine B (anionic) and rhodamine B (zwitterionic)), rhodamine isothiocyanate (RITC)-tagged proteins (bovine serum albumin (BSA), glucose oxidase (GOx) and horseradish peroxidase (HRP)), and a Cy5-tagged single-stranded oligonucleotide (Cy5-*ss*DNA) were readily sequestered within the interior of the preformed hydrated *trans-*azoTAB:PAA particles (Fig. 1d; Supplementary Fig. 5). Typical partition coefficients measured for dyes were in the range of 40–1800 (Supplementary Table S3). Diffusion-mediated uptake of the guest biomacromolecules at concentrations up to 0.05–1 μM occurred without significant change in the morphology or mesostructure of the particles (Supplementary Fig. 6), suggesting that water-soluble and water-insoluble guests could be readily accommodated within the PAA-enriched water domains and *trans-*azoTAB micelles, respectively, of the polyelectrolyte-surfactant matrix. Interestingly, incarceration of large water-soluble biomacromolecules such as HRP in the hydrated *trans-*azoTAB:PAA microparticles occurred with retention of enzyme (peroxidase) activity (Supplementary Fig. 5).

### Blue light-induced dynamic shaping of *trans/cis-*azoTAB:PAA microparticles

**Figure 2:**
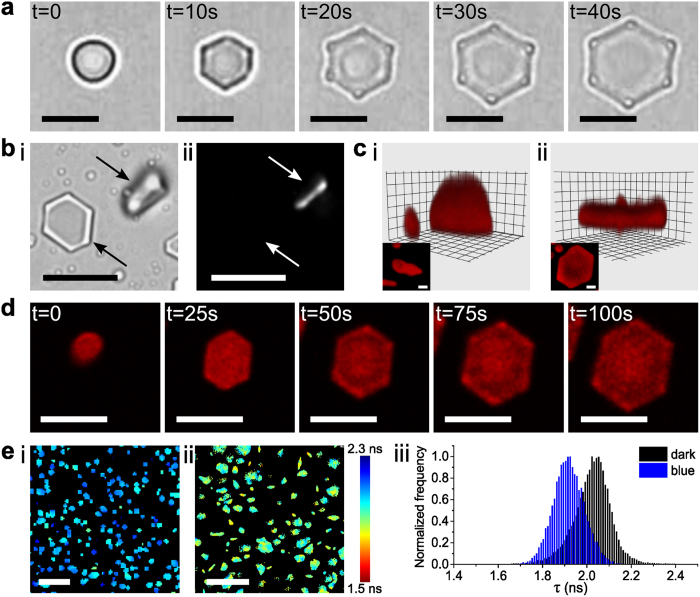
(**a**) Time-lapse series of optical microscopy images showing blue light-induced transformation of a single *trans-*azoTAB:PAA irregular particle mounted on a highly PEGylated glass substrate into a *trans/cis-*azoTAB:PAA hexagonal platelet within 40 s; scale bars = 5 μm (see Supplementary Movie S1). Note the appearance of optically distinct regions at the corners of the hexagonal platelets after 20 s. (**b**) Optical (i) and polarized (ii) microscopy images showing two *trans/cis-*azoTAB:PAA hexagonal platelets oriented either face- or side-on (arrow) with respect to the substrate. Birefringence is only observed when the hexagonal axis is not perpendicular to the substrate; scale bars = 10 μm. (**c**) Confocal microscopy images of a single Nile red-stained *trans-*azoTAB:PAA microparticle (i) before and (ii) after *in situ* blue light irradiation showing formation of a *trans/cis-*azoTAB:PAA hexagonal platelet via lateral expansion and vertical contraction; insets show corresponding top views; scale bars = 1 μm. (**d**) Time-dependent video images showing extensive spreading of a single Cy5-*ss*DNA-containing *trans-*azoTAB:PAA microparticle mounted on a PEGylated glass substrate to produce a well-defined *trans/cis-*azoTAB:PAA hexagonal platelet; scale bars = 5 μm (see Supplementary Movie S2). (**e**) Fluorescence lifetime maps for sulforhodamine B-doped hydrated azoTAB:PAA microparticles mounted on a highly PEGylated glass substrate in the dark (i), or after blue light irradiation (ii); colours map different fluorescence lifetimes; scale bars = 10 μm. (iii) Corresponding fluorescence lifetime distribution histograms extracted from fitting of the FLIM images.

As a step towards non-equilibrium dynamical behaviour, we exploited the simultaneous generation of spatially confined stochastic *cis-trans* isomerisation events at the photo-stationary state (Fig. 1a) to induce blue light-mediated changes in the shape of the polyelectrolyte-surfactant microparticles. Continuous blue light irradiation of the *trans-*azoTAB:PAA microparticles mounted under water onto highly PEGylated glass surfaces (contact angle = 57 ± 1°; prepared by reaction with 3-[methoxy(polyethyleneoxy)propyl] trimethoxysilane for 5 h, see Supporting Methods) transformed the irregular particles plastically into well-defined hexagonal platelets of *trans/cis-*azoTAB:PAA (Fig. 2a; Supplementary Movie 1). A similar morphological transition was observed when an aqueous suspension of *trans-*azoTAB:PAA microparticles was exposed to blue light irradiation (Supplementary Fig. 7). The platelets typically formed on the substrate within 20–40 s of blue light exposure, were 4.7 ± 2.1 μm and 420 ± 40 nm in width and thickness, respectively, and exhibited well defined smooth faces with average interfacial angles of 120.2 ± 0.2° (Supplementary Fig. 8). Optically distinct hemispherical domains were often observed at the corners of the hexagonal platelets (Fig. 2a). Polarized optical microscopy images showed the presence of birefringence when viewed side-on but not along the hexagonal axis (Fig. 2b). Organic dyes and biomolecules sequestered within the *trans-*azoTAB:PAA microparticles were retained in the hexagonal tablets after exposure to blue light (Supplementary Movie 2). Significantly, transformation of the irregular microparticles occurred via lateral spreading of the soft mesophase on the substrate along with vertical contraction (Fig. 2c,d; Supplementary Movie 2), and resulted in preferential alignment of the hexagonal axis parallel to the vertical direction; *i.e*., the azoTAB:PAA cylindrical micelles were preferentially re-oriented perpendicular to the substrate surface. A similar partial re-orientation was observed in SAXS profiles recorded on bulk samples of hydrated *trans*-azoTAB:PAA mounted on mica and exposed to continuous blue light (Supplementary Fig. 9). It should be noted that the morphological changes were induced by non-polarized, randomly oriented and incoherent sources, suggesting a re-orientation mechanism independent of the light polarization pattern. Similar shape transformations were also observed with linearly polarized light.

Based on these observations, we attributed the photo-induced shape transition to softening and fluidization of the hydrated polyelectrolyte-surfactant matrix during transformation to the photostationary *trans/cis* state, which in turn facilitated re-organization of the randomly aligned mesostructure into hexagonal platelets. We confirmed photo-induced softening of the microparticles by fluorescence lifetime imaging microscopy (FLIM) experiments, in which sulforhodamine B was sequestered into the *trans-*azoTAB:PAA microparticles and used as a water-soluble molecular rotor to determine the local micro-viscosity within the hydrated domains before and after blue light irradiation (Fig. 2e). The corresponding lifetimes were 2.03 ± 0.03 ns and 1.92 ± 0.01 ns, respectively, equivalent to a decrease in the local micro-viscosity from 4.1 ± 0.3 to 2.9 ± 0.2 cP on formation of the *trans/cis*-azoTAB:PAA hexagonal platelets. This was consistent with a measured 1.5 MPa decrease in the Young’s modulus of the air-dried *trans/cis-*azoTAB:PAA hexagons compared with an initial value of 13 MPa for the air-dried *trans-*azoTAB:PAA microparticles.

**Figure 3:**
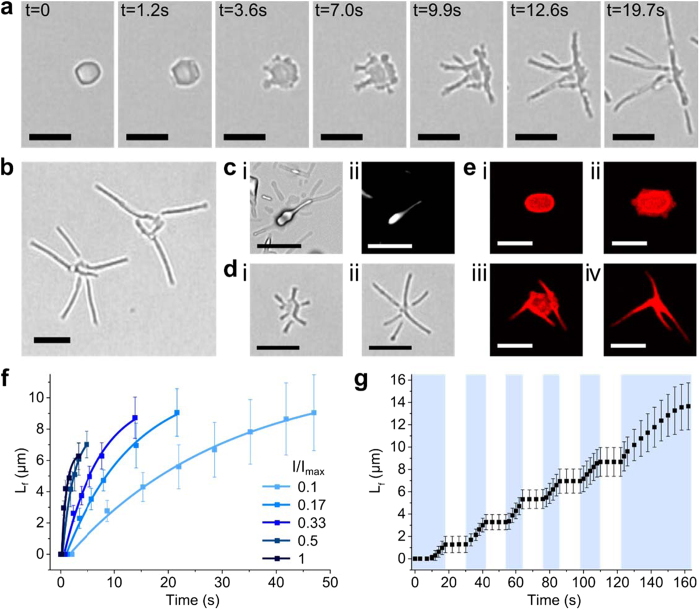
(**a**) Time-lapse series of video images showing blue light-induced transformation of a single *trans*-azoTAB:PAA particle into a multipodal *trans/cis*-azoTAB:PAA micro-architecture within 20 s; scale bars = 5 μm. (**b**) Optical microscopy high magnification image showing two multipodal *trans/cis*-azoTAB:PAA micro-architectures comprising straight-sided filamentous outgrowths connected through a central core, scale bar = 5 μm. (**c**) Optical (i) and corresponding polarized (ii) microscopy images showing birefringence associated with the core and an adjacent filament of a multipodal *trans/cis*-azoTAB:PAA structure, scale bars = 5 μm. (**d**) Optical microscopy images showing simultaneous growth of straight-edged filaments from the corners of a single hexagonal platelet of *trans/cis-*azoTAB:PAA after exposure to blue light for (i) 10 s and (ii) 20 s to produce a discrete multipodal architecture; scale bars = 5 μm. (**e**) Time-lapse series of confocal fluorescence microscopy images of a single *trans-*azoTAB:PAA microparticle containing Cy5-ssDNA before (i) and after exposure to blue light for 10 s (ii), 45 s (iii) and 80 s (iv); scale bars = 5 μm. (**f**) Plots showing time-dependent increases in filament length (L_f_) under blue light (turned on at t = 0) at varying light intensities (I/I_max_). (**g**) Plot showing time-dependent changes in filament length (L_f_) under repeated cycles of dark and blue-light irradiation (marked as white and blue regions, respectively).

Given that microparticle spreading and hexagonal re-alignment of the hydrated azoTAB:PAA cylindrical micelles were influenced by the presence of the highly PEGylated glass surface (contact angle = 57 ± 1°; PEGylation time = 5 h), we investigated the effect of substrate hydrophilicity on the photo-induced shape transition. Remarkably, when the glass substrates were PEGylated for a reduced period of 2 h (contact angle = 62 ± 1°) complex multipodal microarchitectures of *trans/cis-*azoTAB:PAA were spontaneously produced from each microparticle of *trans-*azoTAB:PAA after 10–20 s of exposure to continuous blue light (Fig. 3a; Supplementary Movie 3). The multipodal structures were stable under blue light irradiation and typically consisted of between 2–8 straight-sided filamentous outgrowths that were <400 nm in width, up to 12 μm in length, and randomly arranged around an irregular core (Fig. 3b). Significantly, the width of the filaments seemed effectively unchanged between different multipodal micro-architectures, and was similar in value to the thickness of the hexagonal tablets, suggesting a common structural basis for their mechanism of formation. Polarized light microscopy images indicated that the reconfigured core and apposed regions of the filaments were birefringent (Fig. 3c), although the outer regions of the filaments remained dark, possibly because of their decreased thickness. The filaments were stable up to 5 min after switching off the blue light, after which their edges became less distinct possibly due to wetting of the substrate.

Blue light-induced growth of individual multipodal micro-architectures occurred via the rapid restructuring of single hexagonal platelets of *trans/cis-*azoTAB:PAA that were transiently formed on the substrate by photo-mediated spreading of the irregular *trans-*azoTAB:PAA microparticles. This was confirmed by allowing hexagonal platelets of *trans/cis-*azoTAB:PAA produced in aqueous suspension from blue light exposure of *trans-*azoTAB:PAA microparticles to settle onto a glass surface that had been PEGylated for 2 h; in such cases, multipodal structures were formed on immediate contact with the substrate provided the blue light was on (Supplementary Movie 4; Supplementary Fig. 10). In general, the filaments originated from the (10) side faces and corners of the *trans/cis-*azoTAB:PAA hexagonal platelets (Fig. 3d), and developed independently as single non-interacting fibres by anisotropic spreading of the polyelectrolyte-surfactant core along with sequestered organic dyes and biomolecules (Fig. 3e).

Growth of the filamentous extensions within individual multipodal structures followed an exponential function that depended on the blue light intensity (Fig. 3f). Increases in the final length of the filaments as well as the number of filaments contained within individual multipodal architectures correlated with increases in the initial surface area of the *trans-*azoTAB:PAA particles (Supplementary Fig. 11a), which were consistent with a mechanism based in part on the redistribution of the core material by mass transfer into the thread-like extensions. Moreover, growth of the filaments occurred after a delay time (δ_blue_) of ~0.2–2 s. Since 1/δ_blue_ was proportional to the applied light intensity (Supplementary Fig. 11b), the delay time was attributed to a one-photon azoTAB photo-isomerisation process, consistent with previous reports on azobenzene photo-isomerisation^12^,^39^. In contrast, the inverse of the time constants (1/τ_blue_) associated with filament growth increased exponentially with light intensity, rather than linearly (Supplementary Fig. 11b). This suggested that filament growth was not directly induced by individual photons, but by the interlinked dynamics of photo-isomerisation, anisotropic spreading, and re-organization of the intrinsic structure of the *trans/cis-*azoTAB:PAA assembly. Significantly, filament growth became immediately arrested when the blue light was switched off and was re-established on re-exposure to blue light (Fig. 3g), indicating that formation of the multipodal structure required a continuous throughput of energy.

### UV-induced melting and self-division of azoTAB:PAA mesophases

**Figure 4:**
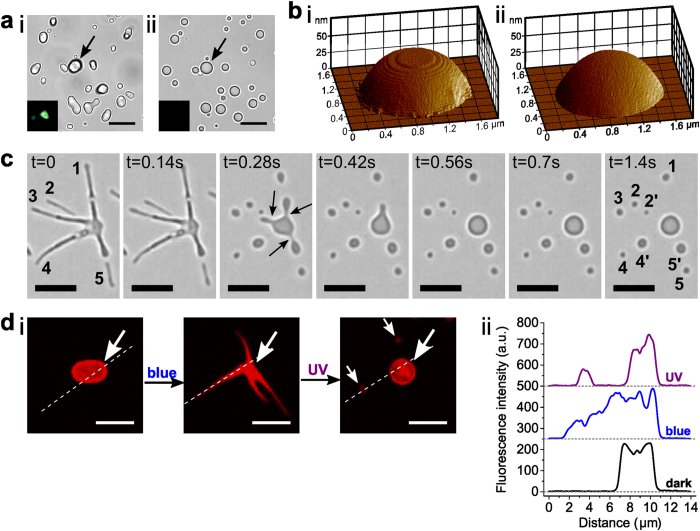
(**a**) Optical microscopy images of *trans-*azoTAB:PAA microparticles (i) before and (ii) during UV-light irradiation to produce single spherical particles of *cis-*azoTAB:PAA; insets show particle highlighted by the arrow under polarized light indicating loss of birefringence after UV exposure; scale bars = 10 μm. (**b**) 3D AFM imaging of a single air-dried *trans*-azoTAB:PAA microparticle viewed (i) in the dark, and (ii) under *in situ* UV irradiation and transformation to *cis-*azoTAB:PAA, showing photo-induced loss of surface texture. (**c**) Time-lapse series of video images showing UV-induced fragmentation and division of a single *trans/cis*-azoTAB:PAA multipodal structure into a series of spherical particles of *cis*-azoTAB:PAA within 1 s. Arrows highlight localised pinching events; numbers correspond to spherical particles produced by fragmentation of marked filaments; scale bars = 5 μm. (**d**) (i) Confocal fluorescence microscopy images of a single azoTAB:PAA microparticle containing Cy5-*ss*DNA during blue/UV light-induced reconfiguration. Arrows highlight the position of the initial particle and products; scale bars = 5 μm. (ii) Corresponding fluorescence intensity profiles recorded along the dotted lines shown in (i).

Experiments were also undertaken in which we replaced the blue light source with continuous UV irradiation to transform the *trans-*azoTAB:PAA particles to a photo-stationary state comprising close to 100% of the *cis-*azoTAB isomer (Fig. 1a). Under these conditions, individual microparticles of *trans-*azoTAB:PAA immediately melted to produce similarly sized single non-birefringent spherical particles of an isotropic *cis-*azoTAB:PAA phase that appeared as hemispheres when mounted under water onto 2- or 5-h PEGylated glass substrates (Fig. 4a; Supplementary Fig. 12; Supplementary Movie 5). The melting process was accompanied by a loss of surface texture (Fig. 4b, Supplementary Fig. 13), a decrease in Young’s modulus of 4.9 ± 1 MPa from an initial value of 13.7 ± 0.7 MPa for the air-dried *trans-*azoTAB;PAA microparticles, and a decrease of the local micro-viscosity from 4.1 ± 0.3 to 1.8 ± 0.2 cP for the hydrated soft solids (Supplementary Fig. 14).

Given these observations, we designed a procedure for the energy-driven transformation of single trans-azoTAB:PAA microparticles into multiple cis-azoTAB:PAA spherical droplets via autonomous fragmentation of multipodal trans/cis-azoTAB intermediates. For this, we mounted trans-azoTAB:PAA microparticles under water onto PEGylated glass substrates (processed for 2 h) and exposed the particles to blue light for around 20 s, followed by UV irradiation for a few seconds. As a consequence, localized clusters of small spherical particles of cis-azoTAB:PAA were produced by UV-induced fragmentation of individual filaments within each of the trans/cis-azoTAB multipodal micro-architectures (Fig. 4c,d; Supplementary Movie 6). Division occurred principally through pinching of the filaments (Fig. 4c), suggesting the onset of an interfacial Rayleigh-Plateau instability arising from the interplay between capillary, viscous and wetting forces^40^. The number of cis-azoTAB:PAA particles formed per filament increased with filament length (Supplementary Fig. S15), and the total number of particles produced increased with the number of filaments per trans/cis-azoTAB:PAA multipodal structure (Supplementary Fig. S15). Interestingly, the above procedure could be used to transfer molecular dyes (Nile red) or biomacromolecules (Cy5-*ss*DNA) trapped within the trans-azoTAB:PAA microparticles to the filaments of the *trans/cis-*azoTAB:PAA multipodal intermediates, followed by further relocation into the *cis*-azoTAB:PAA spherical particles (Fig. 4d; Supplementary Fig. 16). Finally, the robustness of the morphological changes was demonstrated by subjecting the particles to multiple sequential irradiations, which confirmed that the shape transformations to hexagons (Supplementary Movie S7) or to multipodal architectures (Supplementary Movie S8), as well as the back-conversion to droplets and self-division into smaller droplets, were reversible and repeatable.

## Discussion

**Figure 5:**
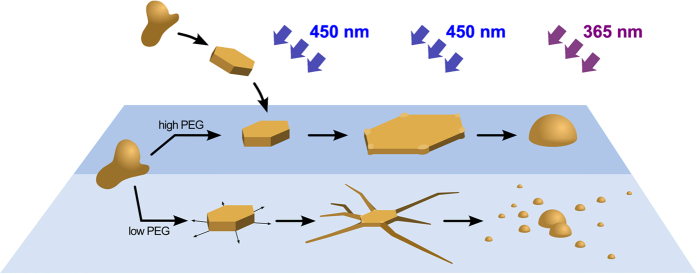
Graphic showing summary of light-induced dynamic shaping and division of azoTAB:PAA microarchitectures via substrate-mediated azobenzene photomechanics. Irregular microparticles of *trans-*azoTAB:PAA (left side) in suspension or mounted on PEGylated glass substrates transform into hexagonal platelets of *trans/cis-*azoTAB:PAA under blue light. The latter reconfigure into multipodal microarchitectures of *trans/cis-*azoTAB:PAA on substrates of lower hydrophilicity. UV irradiation transforms the hexagonal platelets and multipodal structures into single hemispherical particles or clusters of *cis-*azoTAB:PAA, respectively.

Our results indicate that irregularly shaped micrometre-sized particles of the polyelectrolyte-surfactant complex *trans-*azoTAB:PAA can be readily prepared in water as a hydrated soft solid consisting of a hexagonally packed arrangement of cylindrical azoTAB micelles decorated with PAA chains and water molecules. The hydrated microparticles spontaneously sequester a wide range of fluorescent molecular dyes, enzymes and single-stranded oligonucleotides without significant change in mesostructure, and provide a host matrix for enzyme activity. Significantly, by using non-covalent interactions and a hydrated particle-confined soft matrix we are able to induce dynamic shaping and autonomous division of multipodal polyelectrolyte-surfactant microarchitectures via substrate-mediated azobenzene photomechanics, as summarized in Fig. 5.

Generation of the photostationary *trans/cis* state by irradiation of an aqueous suspension of *trans-*azoTAB:PAA with continuous blue light leads to softening and increased fluidity of the microparticles. The onset of fluidization is consistent with previous reports on UV-induced amorphization of azobenzene-based polymer films^17^, and softening in pure crystals of azobenzene molecules^10^,^41^,^42^,^43^,^44^ and azobenzene-doped elastomers^28^,^31^,^45^, which has been attributed to the higher free volume requirements of the *cis* bent conformation compared with the *trans* isomer^27^,^46^. We therefore postulate that blue light-induced fluidization facilitates the co-alignment of kinetically-trapped, randomly arranged mesostructured domains in the *trans*-azoTAB:PAA particles such that the irregular particles of *trans*-azoTAB:PAA are reconfigured into hexagonal platelets with well-defined low energy faces. This mechanism is consistent with the results of experiments in which we replaced the relatively short-chain PAA (M_w_ = 5.1 kDa) with a high molecular weight PAA (M_w_ = 450 kDa). Although irregular particles of *trans*-azoTAB:PAA were produced, these did not transform into hexagonal platelets when exposed to blue light even after 200 s, suggesting that increasing the level of polyelectrolyte entanglement within the interstitial spaces of the hexagonally packed cylindrical azoTAB micelles increased the kinetic stability of the as-prepared polyelectrolyte-surfactant mesophase.

The mechanisms responsible for cooperative motion at length scales considerably larger than the size of the surfactant or polyelectrolyte chains remain to be elucidated. Energization of the system with continuous, non-polarized blue light produces oscillating populations of *trans* and *cis* azoTAB isomers, driving motion at the molecular scale (bending/unbending), which when spatially confined within the mesostructured domains of the microparticles generates a collective response, as recently reported in azobenzene-based supramolecular nanofibers^47^, eventually leading to mechano-transduction at the micrometre length scale. In this respect, previous studies on photo-isomerization in azobenzene-modified liquid crystal polymers indicate that the corresponding reduction in order parameter on formation of the all-*cis* state gives rise to anisotropic contractions/expansions in the material^27^. Thus, it seems feasible that the entropic penalty for the inclusion of the bent *cis* isomer within cylindrical micelles of hydrated *trans*-azoTAB:PAA generates an internal stress field and surface instabilities that can be dissipated through favourable interactions with an underlying solid substrate. In this regard, the *trans/cis*-azoTAB:PAA hexagonal tablets often displayed small hemispherical domains of different optical density (Fig. 2a), suggesting the onset of localized fluidization at their high surface energy corners that results in the substrate-mediated outward flow of the mesophase into thread-like extensions. The anisotropic spreading process is linked to the dynamics of initial azoTAB photo-isomerisation (<2 s delay time) and is dependent on the presence of continuous photo-isomerisation events, suggesting that on-going generation of a stress field via photo-activated *trans/cis* oscillations in the core of the particles is required to produce the multipodal structures. This internally derived mechanism was consistent with the observation that filament growth and formation of the multipodal structures were observed when excess azoTAB in the supernatant was replaced with pure water after adhesion of the *trans*-azoTAB:PAA particles to a 2-h PEG-treated substrate. Interestingly, a 4-fold decrease in the rate of filament growth was observed under these conditions or when the supernatant was replaced with an aqueous PAA solution, indicating that propagation of the filaments over the substrate was also facilitated by azoTAB molecules present in free solution.

The highly anisotropic flow process and formation of filaments of uniform width suggest an underlying structural mechanism related to the height of the side faces of the hexagonal platelets – *i.e.* the dimensions of the optically distinct hemispherical corner domains – as well as re-orientation of the cylindrical micelles parallel to the substrate. Although the latter process requires further experimental confirmation, we note that the core and filaments exhibit birefringence, whereas the hexagonal platelets are dark when oriented along their principal axis, and UV-induced transformation of the filaments obliterates the anisotropic morphology to produce clusters of non-birefringent *cis*-azoTAB:PAA spherical particles. The latter process occurs via UV-induced self-division along with retention of functional molecules within the polyelectrolyte-surfactant matrix, suggesting a future route to the design of new types of adaptive hybrid materials based on the self-assembly and azobenzene photomechanics of polyelectrolyte-surfactant microparticles.

## Methods

### Preparation of *trans*-azoTAB:PAA microparticles

A 50 mM aqueous stock solution of azoTAB was prepared by dissolving a given amount of synthesized azoTAB powder (see Supporting Methods for synthesis details) in milli-Q water, then adjusting the pH to 8 with 0.1 M NaOH or HCl solutions. The solution was stored in the dark for 3 days prior use to ensure equilibration to 100% *trans*-azoTAB isomers. *Trans*-azoTAB:PAA microparticles were prepared in water by addition of an aliquot of a 50 mM PAA (M_w_ = 5,100 g.mol^−1^) aqueous stock solution at pH 8 to a sub-cmc azoTAB solution (final [azoTAB] = 5 mM, final azoTAB:PAA molar ratio of 2:1). Similar preparations undertaken at charge ratios of 1:1 or with excess PAA produced nanoparticles of *trans*-azoTAB:PAA.

### Molecular uptake in *trans*-azoTAB:PAA microparticles

Aliquots of 0.1 mM stock solutions of fluorescent dyes or fluorescently labelled biomacromolecules were added to a freshly prepared suspension of *trans*-azoTAB:PAA microparticles to give a final guest molecule concentration of 0.05–1 μM. Sequestration within the microparticles was assessed by epifluorescence or confocal microscopy. Dyes with excitation wavelengths >600 nm were used to limit azoTAB photo-isomerisation during the fluorescent imaging of the particles.

### Determination of HRP enzymatic activity within *trans*-azoTAB:PAA microparticles

1 μL of a 5 μM stock solution of HRP in water was added to 97 μL of a 2:1 mol/mol *trans-*azoTAB:PAA suspension ([azoTAB] = 5 mM, final [HRP] = 50 nM), followed by addition of 1 μL of a 0.1 mM stock solution of Amplex Red in DMSO (final [Amplex Red] = 1 μM). Just before loading the particle suspension into PEG-functionalized capillary slides, 1 μL of a freshly prepared 0.1 mM stock solution of H_2_O_2_ in water was added to initiate the reaction (final [H_2_O_2_] = 1 μM). Particles were imaged over time by confocal microscopy using an excitation wavelength of 561 nm to excite the product (resorufin) of the HRP-mediated oxidation of Amplex Red. Control experiments were performed in the absence of HRP.

### Light-induced morphological transformations

Photo-induced changes in microparticle morphology were induced *in situ* in an optical microscope by using a 120 W short-arc Hg light source (OSRAM Licht AG, Germany) equipped with a manual shutter and bandpass filters to select the desired wavelength (blue: 450 < λ < 490 nm, UV: 340 < λ < 380 nm). Hexagonal or filamentous spreading of the microparticles was observed under blue light on glass slides prepared by incubation in PEG solutions for 5 or 2 h, respectively (see below). The average height of the microparticles was calculated by dividing the total volume of the particles (derived from the radius of UV-irradiated spherical droplets) by the projected area of the hexagonal profile measured in ImageJ.

In other experiments, a suspension of *trans*-azoTAB:PAA microparticles was loaded onto 2-h PEG-functionalized glass capillary slides, then subjected to blue light irradiation on the optical microscope for 20–40 s. After formation of the multipodal structures, the blue light was turned off and the samples were irradiated with UV light for 1–5 s, resulting in filament reconfiguration. A similar procedure was monitored by confocal microscopy imaging in which the light-induced morphological changes were performed by using the built-in 50 mW diode laser (λ = 405 nm) or 100 mW Ar laser (λ = 458 nm).

### Glass slide functionalization

The PEG-functionalization of glass slides was performed by incubation of ethanol-rinsed glass coverslips (0.13–0.17 mm thick) for 2 hours or 5 hours into a 10 mL toluene solution containing 500 μL of 3-[methoxy(polyethyleneoxy)propyl]trimethoxysilane, resulting in two different levels of PEG coverage. Coverslips where subsequently rinsed with ethanol and water, and dried with compressed air. The surface functionalization was assessed through measurement of water contact angles and surface roughness via PeakForce AFM.

### Fluorescence lifetime microscopy imaging (FLIM) and micro-viscosity measurements

Sulforhodamine B was sequestered into *trans*-azoTAB:PAA microparticles and used as a molecular rotor to determine the fluorescence lifetimes within the hydrated domains before and after blue light or UV irradiation (see Supporting Methods for more details). Typically, 1 μL of a 0.05 mM stock solution of sulforhodamine B in ethanol was added to 99 μL of a 2:1 mol/mol *trans-*azoTAB:PAA suspension (5 mM [azoTAB]) just before fluorescence imaging (final dye concentration, 50 nM). Approximately 5 μL of the dye-doped suspension of microparticles were mounted into PEG-functionalized capillary glass slides, and kept in the dark or irradiated with either blue or UV light for ~1–2 min by using the built-in 50 mW diode laser (λ = 405 nm) or 100 mW Ar laser (λ = 458 nm), after which the light was turned off and lifetime decays were acquired for ~120 s. Acquisition was performed at 25 °C on a Leica TCS SP8 system attached to a Leica DMi8 inverted microscope (Leica Microsystems).

### Optical microscopy

As the *trans*-azoTAB:PAA microparticles tended to wet the surface of untreated glass coverslips, poly(ethylene glycol) (PEG)-functionalized glass substrates were used for optical microscopy imaging. Optical microscopy was performed on a Leica DMI3000 inverted microscope using a ×63 oil immersion lens, 1.4 NA. Polarized light images were acquired on the same microscope equipped with polarisers. Approximately 2.5 μL of the microparticle suspension were mounted into PEG-functionalized capillary glass slides and observed after 5 minutes of equilibration time. Images of the microparticles were recorded on fields of view containing groups of 20–100 particles. Two to three fields of view were recorded for each experiment. Experiments were repeated at least three times. Images were analysed using ImageJ. The circularity (Ψ) of the particles was estimated from Ψ = d_min_/d_max_ where d_min_ and d_max_ are the perimeters of the minimum and maximum circumscribed circles associated with each particle^48^.

### Confocal microscopy

Confocal microscopy imaging was performed using a Leica SP5-II laser scanning microscope attached to a Leica DMI6000 inverted epifluorescence microscope and equipped with a ×63 oil immersion lens, 1.4 NA. Images of the microparticles were recorded on fields of view containing groups of 5–20 particles. Two to three fields of view were recorded for each experiment. Experiments were repeated at least three times. Approximately 95% of the stained droplets were consistent with the images shown in the main text.

### Small angle X-ray scattering

SAXS was undertaken using a Ganesha small angle scattering instrument (SAXSLAB, Denmark) with copper K_α_ radiation and a sample detector distance of 420 mm. The scattering vector (*Q*) scale was calibrated using a silver behenate standard sample^49^. Bulk hydrated *trans*-azoTAB:PAA samples were obtained by sedimentation of the microparticles in a glass vial in the dark. The process was repeated several times by removing the supernatant and adding a freshly prepared suspension of *trans*-azoTAB:PAA particles to accumulate enough material for analysis. The sample was sealed between two mica windows before acquisition. The minimum domain size of the hexagonal mesophase was determined from the width of the Bragg peaks using the Scherrer equation. *In situ* blue light irradiation was achieved by a PCB-mounted LED (Thorlabs, Inc.) operating at 450 ± 9 nm (model M450D3), adapted on a custom-made heat sink and controlled by a T-Cube LED driver (Thorlabs, Inc.), at an intensity of ~20 mW. cm^−2^ (see Supplementary Table 2).

### Atomic force microscopy

AFM was performed on a MultiMode VIII microscope with Nanoscope V controller, using PeakForce feedback control. A fast-scan module was utilized in combination with SCANASYST-HR cantilevers of nominal tip radius 2 nm (Bruker, CA, USA) to allow visualization of surface morphology on timescales relevant to tracking morphological changes within the sample. The particle suspension was deposited for ~1 min on the cleaved mica followed by removal of the excess supernatant before investigation in ambient conditions. *In situ* light irradiation was achieved by a PCB-mounted LED (Thorlabs, Inc.) operating at 365 ± 4.5 nm (model M365D2) or 450 ± 9 nm (model M450D3), and adapted on a custom-made heat sink and controlled by a T-Cube LED driver (Thorlabs, Inc.), at an intensity of ~10–20 mW. cm^−2^ (see Supplementary Table 2).

The Young’s modulus of the azoTAB:PAA microparticles was measured at the atomic scale by quantitative nano-mechanical measurements using PeakForce microscopy with a multi-mode VIII microscope with Nanoscope V controller. Measurements in ambient environment were conducted with a fast scan head unit and SCANASYST-AIR cantilevers, maximum tip radius 10 nm, and nominal spring constant 0.4 N/m. For measurements in liquid environments, a suspension of microparticles was deposited on cleaved mica and left to stand for ~1 h to allow sedimentation of the particles. The sample was then placed in a liquid cell and measurements were performed with SCANASYST-FLUID cantilevers, nominal tip radius 10 nm and spring constant 0.7 N/m. The system was calibrated using the recommended relative method and Young’s modulus data was extracted via the fitting of measured force curves with a Hertzian DMT model. Deformation of the sample was typically maintained at around 5–6 nm.

## Additional Information

**How to cite this article**: Martin, N. *et al*. Light-induced dynamic shaping and self-division of multipodal polyelectrolyte-surfactant microarchitectures via azobenzene photomechanics. *Sci. Rep.* **7**, 41327; doi: 10.1038/srep41327 (2017).

**Publisher's note:** Springer Nature remains neutral with regard to jurisdictional claims in published maps and institutional affiliations.

## Supplementary Material

Supporting Information

Movie S1

Movie S2

Movie S3

Movie S4

Movie S5

Movie S6

Movie S7

Movie S8
