# Supplementary material for: Light-induced dynamic shaping and self-division of multipodal polyelectrolyte-surfactant microarchitectures via azobenzene photomechanics
Source: Sci Rep. 2017 Jan 23;7:41327. doi: 10.1038/srep41327 (PMC5256308; doi:10.1038/srep41327)
Supplement: Supporting Information [file srep41327-s1.pdf]

Supporting Information: Movies, Materials and Methods, and Supplementary Figures

Light-induced dynamic shaping and self-division of multipodal polyelectrolyte-surfactant microarchitectures via azobenzene photomechanics

Nicolas Martin[1], Kamendra P. Sharma[1,2], Robert L. Harniman[1], Robert M. Richardson[3], Ricky J. Hutchings[1], Dominic Alibhai[4], Mei Li[1], Stephen Mann[1,*]

[1] Centre for Organized Matter Chemistry, School of Chemistry, University of Bristol, Bristol BS8 1TS, UK

[2] Department of Chemistry, Indian Institute of Technology Bombay, Mumbai, 400076, India

[3] School of Physics, H. H. Wills Physics Laboratory, University of Bristol, Tyndall Avenue, Bristol BS8 1TL, UK

[4] Wolfson Bioimaging Facility, Faculty of Biomedical Sciences, University of Bristol, University Walk, Bristol BS8 1TD, UK

* Correspondence to s.mann@bristol.ac.uk

1. Movies

Movie S1
Optical microscopy video showing blue light-induced transformation of *trans*-azoTAB:PAA irregular microparticles into hexagonal platelets of *trans/cis*-azoTAB:PAA. Samples were mounted under water on 5-h PEG-reacted glass slides. Movie is shown at 3 times of real-time speed at 21 frames per second. Total duration of recording was 42 seconds in real time.

Movie S2
Confocal fluorescence microscopy video (excitation wavelength = 633 nm, emission recorded in the range 650-800 nm) showing blue light-induced spreading of Cy5-*ss*DNA-doped *trans*-azoTAB:PAA microparticles into hexagonal platelets of *trans/cis*-azoTAB:PAA with retention of the guest oligonucleotide molecules. Samples were mounted under water on 5-h PEG-reacted glass slides. Movie is shown at 10 times of real-time speed at 10 frames per second. Total duration of recording was 103 seconds in real time.

Movie S3
Optical microscopy video showing blue light-induced transformation of *trans*-azoTAB:PAA irregular microparticles into multipodal micro-architectures of *trans/cis*-azoTAB:PAA filaments. Samples were mounted under water on 2-h PEG-reacted glass slides. Movie is shown at 3 times of real-time speed at 21 frames per second. Total duration of recording was 20 seconds in real time.

Movie S4
Optical microscopy video showing hexagonal platelets of *trans/cis*-azoTAB:PAA produced in aqueous suspension from blue light exposure of *trans*-azoTAB:PAA microparticles, followed by attachment onto a 2 h-PEG-reacted glass surface and immediate transformation under blue light to produce multipodal structures. No filament growth is observed for hexagonal platelets that remain in suspension. Movie is shown at 3 times of real-time speed at 21 frames per second. Total duration of recording was 38 seconds in real time.

Movie S5
Optical microscopy video showing UV-induced transformation of irregular *trans*-azoTAB:PAA particles to circular *cis*-azoTAB:PAA droplets. Movie is shown at 2 times of real-time speed at 14 frames per second. Total duration of recording was 10 seconds in real time.

Movie S6
Optical microscopy video showing blue light-induced transformation of *trans*-azoTAB:PAA irregular microparticles into multipodal micro-architectures of *trans/cis*-azoTAB:PAA filaments spread across a 2-h PEG-reacted glass substrate, followed by UV-induced division of the filaments into localized clusters of small circular particles of *cis*-azoTAB:PAA. Movie is shown at 2 times of real-time speed at 14 frames per second. Total duration of recording was 27 seconds in real time.

Movie S7
Optical microscopy video showing multiple sequential blue-then-UV-light irradiations of *trans*-azoTAB:PAA microparticles and their reversible and repeated transformation between *trans/cis*-azoTAB:PAA hexagonal platelets and *cis*-azoTAB:PAA droplets. Samples were

mounted under water on 5-h PEG-reacted glass slides. Movie is shown at 10 times of real-time speed at 70 frames per second. Total duration of recording was 190 seconds in real time.

Movie S8

Optical microscopy video showing multiple sequential blue-then-UV-light irradiations of *trans*-azoTAB:PAA microparticles and their reversible and repeated transformation between *trans/cis*-azoTAB:PAA multipodal microarchitectures and self-divided *cis*-azoTAB:PAA droplets. Samples were mounted under water on 2-h PEG-reacted glass slides. Movie is shown at 10 times of real-time speed at 70 frames per second. Total duration of recording was 180 seconds in real time.

2. Materials and Methods

Materials

Poly(sodium acrylate) (PAA) with a chain length of ca. 54 monomers (M_w = 5,100 g.mol^{-1}) was purchased from Sigma-Aldrich and used as received. Azobenzene trimethylammonium bromide (azoTAB) was synthesized according to the procedure described by Hayashita *et al.*[1] by azocoupling *p*-ethoxyaniline with phenol, followed by alkylation with dibromoethane then quaternisation with trimethylamine (see below for details). 3-[methoxy(polyethyleneoxy) propyl]trimethoxysilane; 90%, 6-9 PE units was purchased from abcr GmbH, Gute Chemie (Germany). Fluorescent dyes (rhodamine B, sulforhodamine B, nile red, methylene blue, rhodamine isothiocyanate (RITC)), bovine serum albumin (BSA), glucose oxidase from *Aspergillus Niger* (GOx), horseradish peroxidase (HRP), H_2O_2, p-ethoxyaniline, sodium nitrite, phenol, 1,2-dibromoethane, potassium carbonate, potassium iodide and 33% trimethylamine solution in ethanol were purchased from Sigma-Aldrich and used as received. were purchased from Sigma-Aldrich. Cy5-tagged single-stranded oligonucleotide (Cy5-ssDNA) containing 23 bases was purchased from Eurofins Genomics. Amplex Red was purchased from ThermoFischer.

Synthesis of azoTAB

Synthesis of 4-ethoxy-4'-hydroxy-azobenzene (azoH)

Concentrated HCl (17 mL) and ice (80 g) were added to an 1:1 v/v ethanol:water solution (160 mL) containing p-ethoxyaniline (10.3 mL, 80 mmol, 1 equiv.) and sodium nitrite (5.5 g, 80 mmol, 1 equiv.) in an ice bath (T = 0°C). The mixture was stirred for 1h. Cold water (42 mL) containing phenol (7.5 g, 80 mmol, 1 equiv.) and NaOH (6.4 g, 160 mmol, 2 equiv.) was then carefully added to the solution and the mixture was stirred for another 90 min by keeping the temperature below 5°C. The pH of the solution was then adjusted to 1 with concentrated HCl and left to stand for 30 min. The resulting precipitate was filtered, thoroughly washed with water and dried under vacuum overnight to give 4-ethoxy-4'-hydroxy-azobenzene (azoH) as a dark brown powder (76% yield). ^{1}H NMR (400 MHz, $CDCl_3$): δ = 7.86 (d, ^3J(H-H) = 8 Hz, 2H; Ar-H), 7.82 (d, ^3J(H-H) = 8 Hz, 2H; Ar-H), 6.99 (d, ^3J(H-H) = 8 Hz, 2H; Ar-H), 6.94 (d, ^3J(H-H) = 8 Hz, 2H; Ar-H), 4.12 (q, ^3J(H-H) = 6 Hz, 2H; CH_2), 1.46 ppm (t, ^3J(H-H) = 4 Hz, 3H; CH_3); ^{13}C NMR (400 MHz, $CDCl_3$): δ = 161.0 (Ar-C), 157.9 (Ar-C), 147.1 (Ar-C), 146.8 (Ar-C), 124.6 (Ar-C), 124.4 (Ar-C), 115.8 (Ar-C), 114.7 (Ar-C), 63.8 (CH_2O), 14.8 ppm (CH_3); MS (ESI): *m/z*: calcd for $C_{14}H_{14}N_2O_2$: 242.3 $[M]^+$; found: 243.1

Synthesis of 4-ethoxy-(4'-(2-bromoethyloxy)phenyl)azobenzene (azoBr)

A mixture of 4-ethoxy-4'-hydroxy-azobenzene (2.4 g, 10 mmol, 1 equiv.), 1,2-dibromoethane (5.6 g, 3 equiv.), potassium carbonate (2.07 g, 1.5 equiv.) and potassium iodide (0.083 g, 0.05 equiv.) were refluxed in 50 mL of butanone for 48 h in the dark. The reaction mixture was filtered hot to remove solid impurities and salt, and the residue was washed with butanone. The filtrate was collected and the solvent was removed under reduced pressure. The obtained solid was dissolved in dichloromethane (20 mL) and extractions were performed with NaOH

solution (1M, 2 × 8 mL) then pure water (2 × 8 mL). The organic phase was dried with $MgSO_4$ and concentrated. The crude product was recrystallized with hot filtration from ethanol and dried under vacuum to give 4-ethoxy-(4'-(2-bromoethyloxy)phenyl)azobenzene (azoBr) as an orange powder (54% yield). ^{1}H NMR (400 MHz, $CDCl_3$): δ = 7.92 (q, ^3J(H-H) = 8.2 Hz, 4H; Ar-H), 7.00 (dd, ^3J(H-H) = 8.6 Hz, 4H; Ar-H), 4.37 (t, ^3J(H-H) = 8 Hz, 2H; CH_2O), 4.12 (q, ^3J(H-H) = 6 Hz, 2H; CH_2O), 3.67 (t, ^3J(H-H) = 7 Hz, 2H; CH_2Br), 1.46 ppm (t, ^3J(H-H) = 6 Hz, 3H; CH_3); ^{13}C NMR (400 MHz, $CDCl_3$): δ = 161.5 (Ar-C), 160.2 (Ar-C), 146.8 (Ar-C), 146.3 (Ar-C), 124.8 (Ar-C), 124.6 (Ar-C), 114.9 (Ar-C), 114.8 (Ar-C), 68.0 (CH_2O), 63.9 (CH_2O), 28.8 (CH_2Br), 14.8 ppm (CH_3); MS (ESI): *m/z*: calcd for $C_{16}H_{17}N_2O_2BrNa$: 372.2 g.mol^{-1} [M+Na$^+$]$^+$; found: 373.0

Synthesis of azobenzene trimethylammonium bromide (azoTAB)

1 g of 4-ethoxy-(4'-(2-bromoethyloxy)phenyl)azobenzene (4.4 mmol, 1 equiv.) was dissolved in 80 mL of dry THF, followed by the addition of a 33% solution of trimethylamine in ethanol (4.2 mL, 11.5 mmol, 4 equiv.). The mixture was stirred for 6 days in the dark. The resulting precipitate was filtered, washed with THF, and dried under vacuum. The crude product was recrystallized twice from ethanol and dried under vacuum overnight to give azobenzene trimethylammonium bromide (azoTAB) as an orange powder (36% yield). ^{1}H NMR (400 MHz, DMSO): δ = 7.86 (d, ^3J(H-H) = 8 Hz, 2H; Ar-H), 7.82 (d, ^3J(H-H) = 8 Hz, 2H; Ar-H), 7.17 (d, ^3J(H-H) = 8 Hz, 2H; Ar-H), 7.08 (d, ^3J(H-H) = 8 Hz, 2H; Ar-H), 4.56 (m, 2H; CH_2O), 4.11 (q, ^3J(H-H) = 6 Hz, 2H; CH_2O), 3.82 (m, 2H; CH_2N), 3.18 (s, 9H; CH_3N), 1.35 ppm (t, ^3J(H-H) = 4 Hz, 3H; CH_3); ^{13}C NMR (400 MHz, DMSO): δ = 161.3 (Ar-C), 159.9 (Ar-C), 147.1 (Ar-C), 146.4 (Ar-C), 124.7 (Ar-C), 124.5 (Ar-C), 115.8 (Ar-C), 115.4 (Ar-C), 64.5 (CH_2O), 64.1 (CH_2N), 62.5 (CH_2O), 53.6 (CH_3N), 15.0 ppm (CH_3); MS (ESI): *m/z*: calcd for $C_{19}H_{26}N_3O_2$: 328.4 g.mol^{-1} [M–Br$^-$]$^+$; found: 328.2

Determination of the *trans*:*cis* isomer composition under UV and blue light

The photo-induced isomerisation of pure *trans*-azoTAB in water was assessed by UV-vis spectroscopy on a PerkinElmer Lambda 750 spectrophotometer, after irradiation with a PCB-mounted LED (Thorlabs, Inc.) operating at 365 ± 4.5 nm (model M365D2) or 450 ± 9 nm (model M450D3), and adapted on a custom-made heat sink and controlled by a T-Cube LED driver (Thorlabs, Inc.) with adjustable power. Optical intensities were measured with a silicon photodetector (model 918D-UV-OD3R, Newport Corporation, USA). Typical intensities of 0.3-20 mW.cm^{-2} were used to induce *trans*-azoTAB photo-isomerisation. The same LEDs were used for *in situ* irradiation in AFM and X-ray scattering experiments (see below).

AzoTAB solutions containing 100% *trans* isomers were obtained after storage in the dark for 3 days. The UV-vis spectrum of the dark-adapted sample did not change over time, confirming that equilibrium was reached. Spectra acquired on the same solution irradiated with UV (λ = 365 nm, 12.4 mW.cm^{-2}) or blue (λ = 450 nm, 20.9 mW.cm^{-2}) light were used to determine the fractions of *trans* and *cis* isomers at the stationary state. Absorbance was considered to vary in proportion to the composition, according to the relationships:

$$\varepsilon^{UV}(\lambda) = x_{trans}^{UV} \times \varepsilon_{trans}(\lambda) + x_{cis}^{UV} \times \varepsilon_{cis}(\lambda) \qquad \text{(Eq. S1)}$$

$$\varepsilon^{blue}(\lambda) = x_{trans}^{blue} \times \varepsilon_{trans}(\lambda) + x_{cis}^{blue} \times \varepsilon_{cis}(\lambda) \qquad \text{(Eq. S2)}$$

where ε^{UV} (resp. ε^{blue}) is the molar absorption coefficient of the UV- (resp. blue-) adapted solution, x_{trans}^{UV} and x_{trans}^{blue} (resp. x_{cis}^{UV} and x_{cis}^{blue}) are the fractions of *trans* (resp. *cis*) isomer in the UV- or blue-adapted sample. Initial x_{trans}^{UV} and x_{trans}^{blue} values were calculated by assuming $\varepsilon_{cis}(\lambda_{min}^{UV}) << \varepsilon_{trans}(\lambda_{min}^{UV})$ where λ_{min}^{UV} is the wavelength at the minimum of absorption of the UV-adapted sample in the range 300 < λ < 450 nm. The spectrum of the *trans* isomer weighed by the as-so calculated x_{trans}^{UV} (resp. x_{trans}^{blue}) value was subtracted from the UV- (resp. blue-) adapted spectrum to give a putative spectrum for the *cis* isomer. An iterative process was used to minimize the difference between the spectra calculated for the *cis* isomer from the UV- and the blue-adapted solutions in the range 300 nm < λ < 450 nm. At the end of the process, the two calculated spectra for the *cis* isomer converged to a single one, resulting in x_{trans}^{UV} and x_{trans}^{blue} values reported in **Table S1**.

Table S1. Fractions of *trans* and *cis* isomers in the UV- or blue-adapted azoTAB aqueous solutions computed from the UV-vis spectra.

	UV	Blue
x_{trans}	4%	58%
x_{cis}	96%	42%

Light intensities of LEDs

Optical intensities of the LED measured with a silicon photodetector along the emission axis at a distance of 50 mm (which is the typical distance used in all experiments), are reported in Table S2 at different driver settings. The maximum values of 12.4 $mW.cm^2$ for the UV LED and 20.9 $mW.cm^2$ for the blue LED were used for azoTAB isomerisation studies in solution and for *in situ* AFM and X-ray scattering experiments.

Table S2. Light intensities ($mW.cm^{-2}$) measured for the UV and blue LED at different input settings.

T-cube driver setting	UV LED 365 nm	Blue LED 450 nm
1	0.28	0.59
2	0.77	1.8
3	1.4	2.7
4	4.3	7.9
5	9.6	17.1
6	12.4	20.9

Fluorescent labelling of proteins

Protein solutions (4 mg mL^{-1}) were prepared by dissolving freeze-dried protein powders in 0.5 M $NaHCO_3$/$NaCO_3$ buffer at pH 9.5. An aliquot of a freshly-prepared anhydrous DMSO solution of rhodamine isothiocyanate (RITC) (10 mg mL^{-1}) was added to the protein solution at a RITC:protein final molar ratio of ca. 20:1. The reaction mixture was kept at room temperature in the dark for 2-4 hours, then purified by size exclusion chromatography using a

Sephadex G-25 resin (Sigma-Aldrich) eluted with milli-Q water. The concentration of the RITC-labelled protein in the collected fractions was determined by UV-visible spectrophotometry according to the relationship: [protein] = (A_{280} – 0.33 × A_{540}) / $\varepsilon_{protein}$, where A_{280} and A_{540} were the absorbances at 280 and 540 nm, 0.33 the correction factor to account for the dye absorption at 280 nm, and $\varepsilon_{protein}$ the extinction coefficient of the protein (0.665 mL mg^{-1} cm^{-1} (BSA), 1.83 mL mg^{-1} cm^{-1} (CAB) and 0.656 mL mg^{-1} cm^{-1} (HRP)). The RITC:protein final molar ratio was determined from the ratio (A_{540} / ε_{RITC}) / ([protein] (mg mL^{-1}) / $M_{protein}$), where ε_{FITC} was the molar extinction coefficient of RITC at 540 nm (80,000 mol^{-1} L cm^{-1}), and $M_{protein}$ the molar mass of the protein (66,500 g mol^{-1} (BSA), 29,000 g mol^{-1} (CAB) and 44,000 g mol^{-1} (HRP)). Typically, the value RITC:protein was *ca.* 3:1.

Fluorescence lifetime microscopy imaging (FLIM) and micro-viscosity measurements

Molecular rotors are fluorophores that show viscosity-dependent competitive radiative and non-radiative decay pathways,[2] where non-radiative decay comes from intramolecular rotation and is therefore hindered in viscous media. The fluorescence lifetimes τ_f are related to the viscosity via the Förster-Hoffmann equation:[3]

$$\log \tau_f = \log \frac{z}{k_r} + \alpha \log \eta$$

where η is the viscosity, z and α are constants, and k_r is the radiative decay constant.

Excitation was provided by a white light laser with a repetition rate of 20 MHz and an acousto-optical beam splitter (AOBS) to select an excitation wavelength of 561 nm. Images were acquired using a ×63 1.2 NA water immersion objective. Fluorescence was detected using a hybrid detector operating in photon counting mode over an emission range of 572 – 700 nm. A notch filter centred on 561 nm minimised any laser scatter into the detector. Time resolved data was acquired through use of a PicoHarp 300 TCSPC module (PicoQuant) controlled through SymPhoTime64 software (PicoQuant). FLIM images were acquired with 256 x 256 pixels and 4096 time bins. Fitting of FLIM images was performed with the FLIMfit software tool developed at Imperial College London.[4] Temporal binning of the fluorescence decays was performed prior to fitting resulting in 256 time bins per decay and an integrated intensity threshold of 50 photons per pixel was applied. The fluorescence lifetime data was fitted on a pixelwise basis to a single exponential model. Calibration was performed by measuring the fluorescence lifetimes of sulforhodamine B in sucrose:water solutions of known viscosities.[2]

1. Hayashita, T.; Kurosawa, T.; Miyata, T.; Tanaka, K.; Igawa, M. Effect of structural variation within cationic azo-surfactant upon photoresponsive function in aqueous solution. *Colloid. Polym. Sci.* **1994,** *272*, 1611-1619.
2. Kuimova, M. K. Mapping viscosity in cells using molecular rotors. *Phys. Chem. Chem. Phys.* **2012,** *14*, 12671-12686.
3. Forster, T.; Hoffmann, G., Die Viskositätsabhängigkeit der Fluoreszenzquantenausbeuten einiger Farbstoffsysteme. *Z. Phys. Chem.* **1971,** *75*, 63.
4. Warren, S. C.; Margineanu, A.; Alibhai, D.; Kelly, D. J.; Talbot, C.; Alexandrov, Y.; Munro, I.; Katan, M.; Dunsby, C.; French, P. M. W. Rapid global fitting of large fluorescence lifetime imaging microscopy datasets. *PLoS ONE* **2013,** *8*, e70687.

3. Supplementary Figures

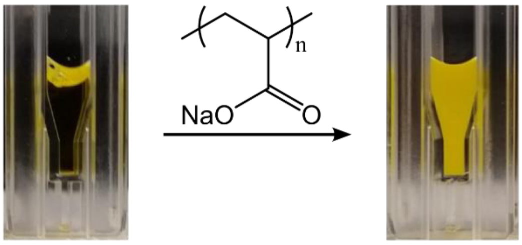

Figure S1. Photographs showing appearance of turbidity in a transparent 5 mM *trans*-azoTAB aqueous solution upon addition of 2.5 mM PAA, attributed to the formation of micrometre-sized hydrated *trans*-azoTAB:PAA particles.

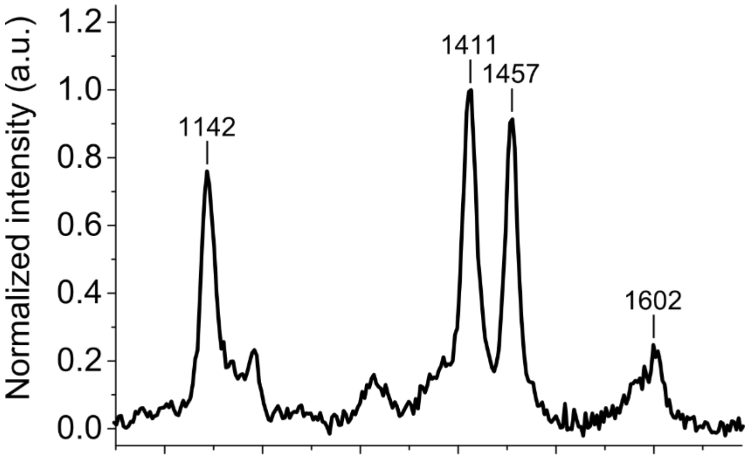

Figure S2. Raman microscopy spectrum of hydrated *trans*-azoTAB:PAA microparticles mounted on a PEG-functionalized glass slide. The peak at 1142 cm^{-1} is assigned to the C-N stretching mode; the peaks at 1411, 1457 and 1602 cm^{-1} are mixed modes that have N=N stretching character in common. [Yoon, J.H.; Yoon, S. *Phys. Chem. Chem. Phys.* **2011**, *13*, 12900-12905]

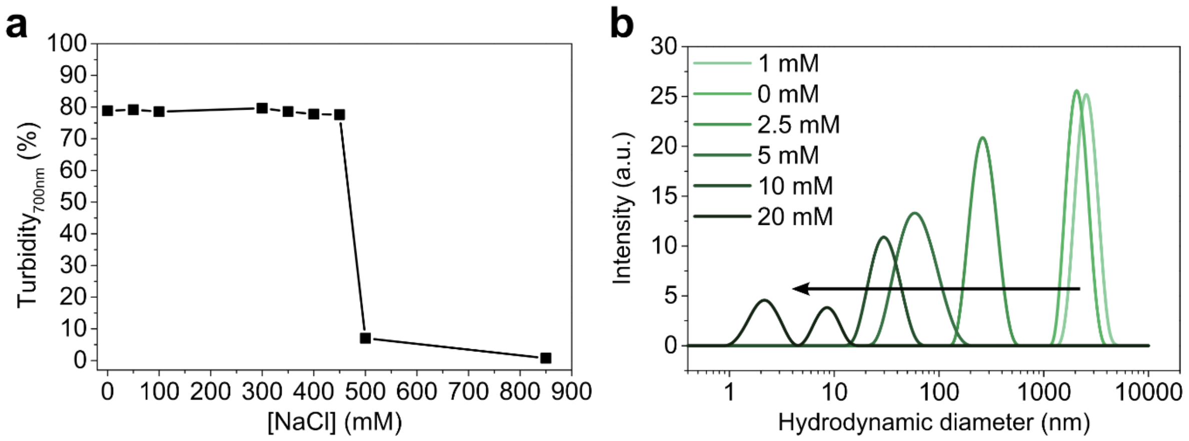

Figure S3. (**a**) Turbidity measured at 700 nm for a 2:1 mol/mol *trans*-azoTAB:PAA suspension at increasing NaCl concentrations showing salt-induced inhibition of particle formation. (**b**) Size distribution of 2:1 mol/mol *trans*-azoTAB:PAA suspensions in the presence of increasing amount of hydroxypropyl β-cyclodextrin (HP-β-CD). HP-β-CD was used as a host molecule for the hydrophobic azobenzene moiety of azoTAB.

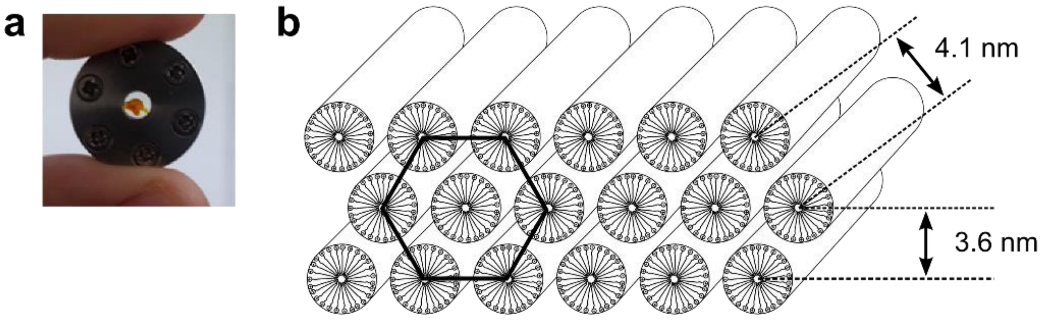

Figure S4. (**a**) Photograph showing bulk hydrated sample of *trans*-azoTAB:PAA prepared by accumulation of sedimented microparticles. (**b**) Schematic representation of the spatial ordering of the hexagonal mesophase derived from X-ray scattering data.

Table S3. Quantification of dye uptake in the *trans*-azoTAB:PAA particles.

Dye	Amount sequestered (%)	Partition coefficient
Methylene blue	17	40
Rhodamine B	38	120
Rhodamine 6G	51	210
Sulforhodamine G	58	280
Sulforhodamine B	86	1200
Nile Red	92	1800

Note: The amount of dye sequestered inside the particles was determined by UV-vis spectroscopy as the percent of dye removed from the supernatant after centrifugation of the suspension of particles compared to the total amount of dye added. In addition, partition coefficients $K = [\text{dye}]_{\text{particles}} / [\text{dye}]_{\text{supernatant}}$ could be derived by estimating the total volume of the azoTAB:PAA mesophase formed per unit volume of aqueous phase. Our estimations give ~5µL of azoTAB:PAA mesophase per mL of aqueous suspension.

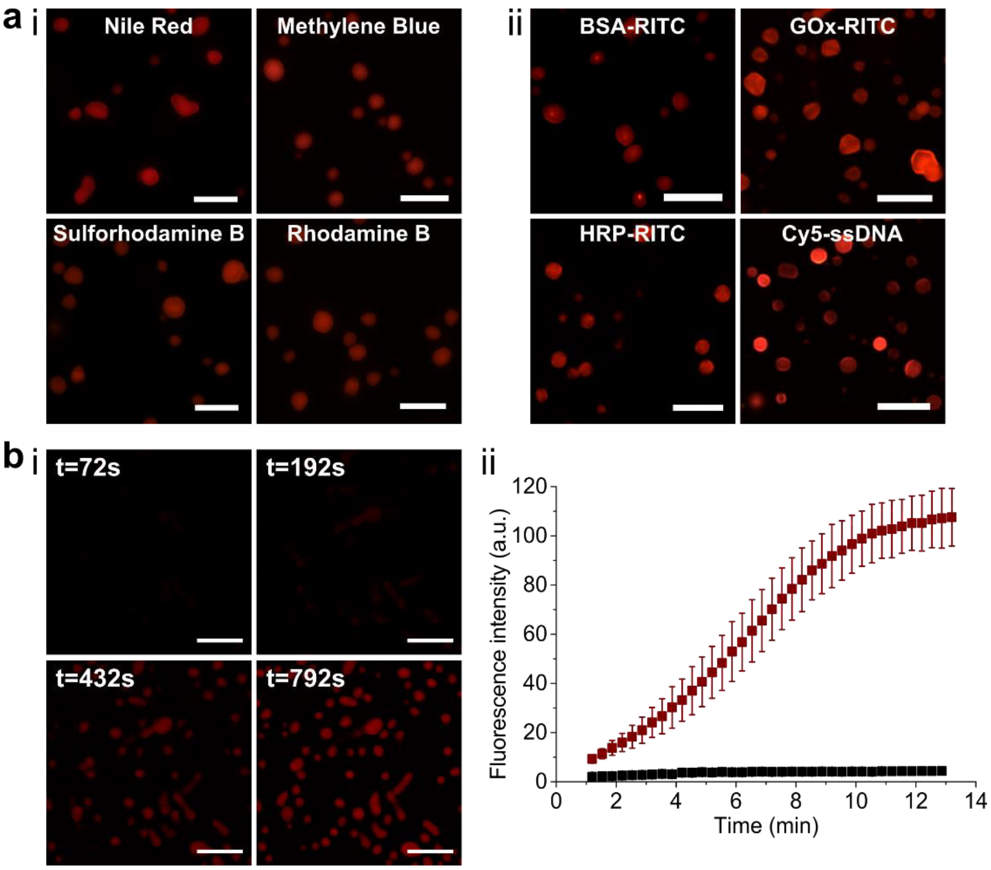

Figure S5. (**a**) Epifluorescence imaging of hydrated *trans*-azoTAB:PAA microparticles in the presence of various fluorescent dyes (i) or fluorescently-labelled biomacromolecules (ii), showing sequestration of the additives in all cases; scale bars = 10 µm. (**b**) (**i**) Time series of confocal fluorescence images of hydrated *trans*-azoTAB:PAA microparticles containing non-fluorescently labelled HRP (50 nM), in the presence of H_2O_2 (1 µM) and Amplex Red (1 µM). The gradual increase in fluorescence intensity over 15 min is associated with the HRP-mediated oxidation of Amplex Red to the fluorescent product resorufin; scale bars = 10 µm. (**ii**) Plot of time-dependent changes in fluorescence intensity of hydrated *trans*-azoTAB:PAA microparticles in the absence (control, black) or presence of HRP (red), confirming the catalytic activity of the incarcerated enzyme.

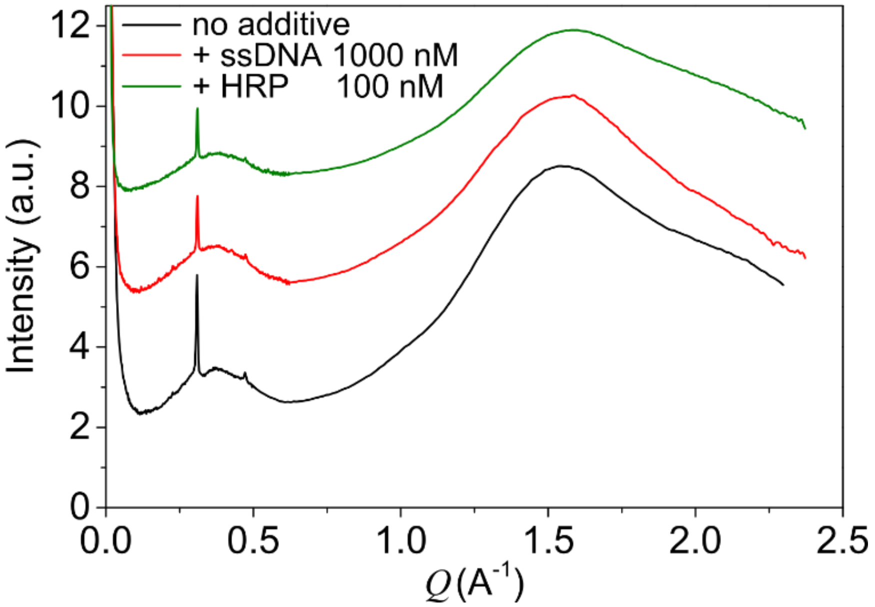

Figure S6. X-ray scattering profiles of hydrated *trans*-azoTAB:PAA containing Cy5-ssDNA or RITC-HRP showing same peaks as in the absence of additives.

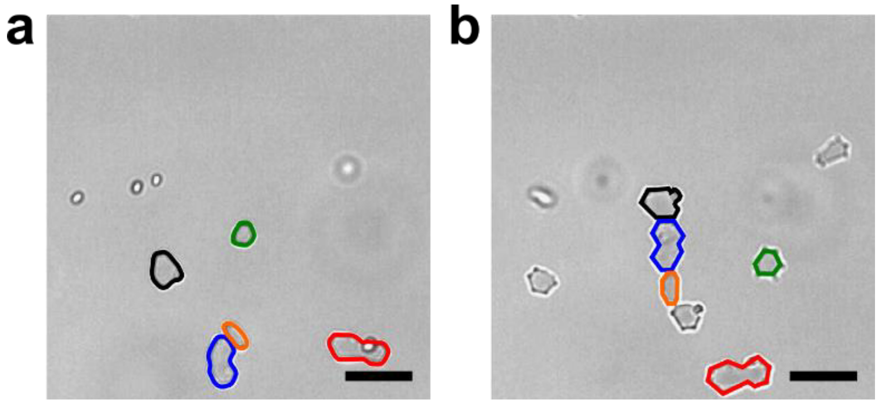

Figure S7. Optical microscopy images of suspended (non-adherent) *trans*-azoTAB:PAA microparticles; (**a**) before and (**b**) under blue light irradiation, showing appearance of hexagonal-like *trans/cis*-azoTAB:PAA particles (average interfacial angle ≈ 120°). Particles are moving in suspension and delineated with coloured lines to identify them before and during irradiation; scale bars = 10 μm.

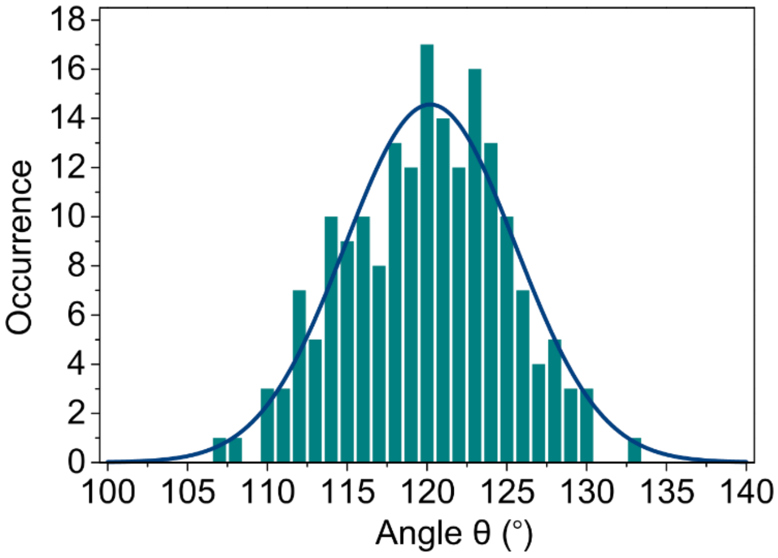

Figure S8. Angle distribution between adjacent facets in *trans/cis*-azoTAB:PAA hexagonal platelets.

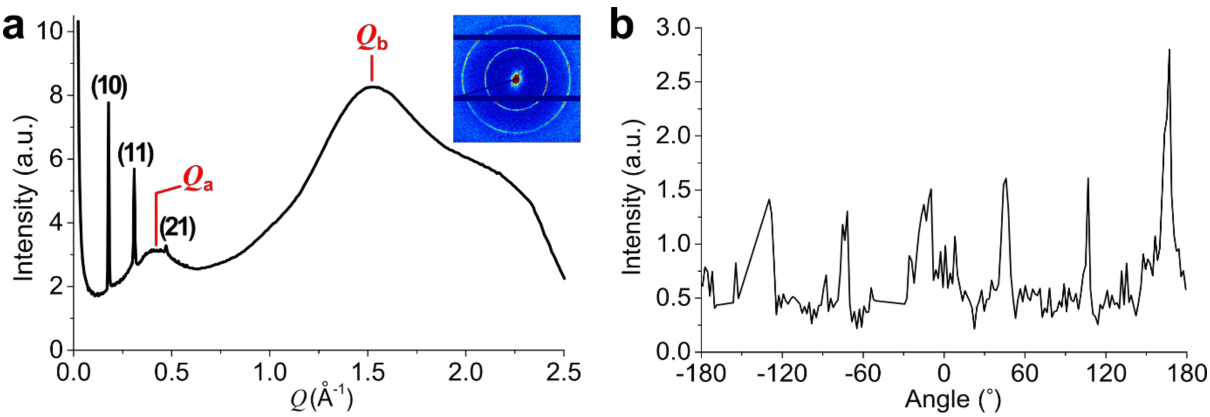

Figure S9. (**a**) X-ray scattering profile of hydrated bulk *trans/cis*-azoTAB:PAA under continuous blue light irradiation, showing three low angle Bragg reflections associated with a 2D hexagonal mesostructure (0.178 Å^{-1} (10), 0.309 Å^{-1} (11) and 0.471 Å^{-1} (21), and broad peaks at Q_a = 0.421 Å^{-1} and Q_b = 1.518 Å^{-1}. The presence of the (10) reflection suggests partial re-orientation of the hexagonal axis perpendicular to the substrate. Inset shows 2D ring pattern with superimposed hexagonal spots. (**b**) Azimuthal scan showing six intensity peaks separated by 60° representing the spots on the hexagonal pattern shown in the inset in (**a**).

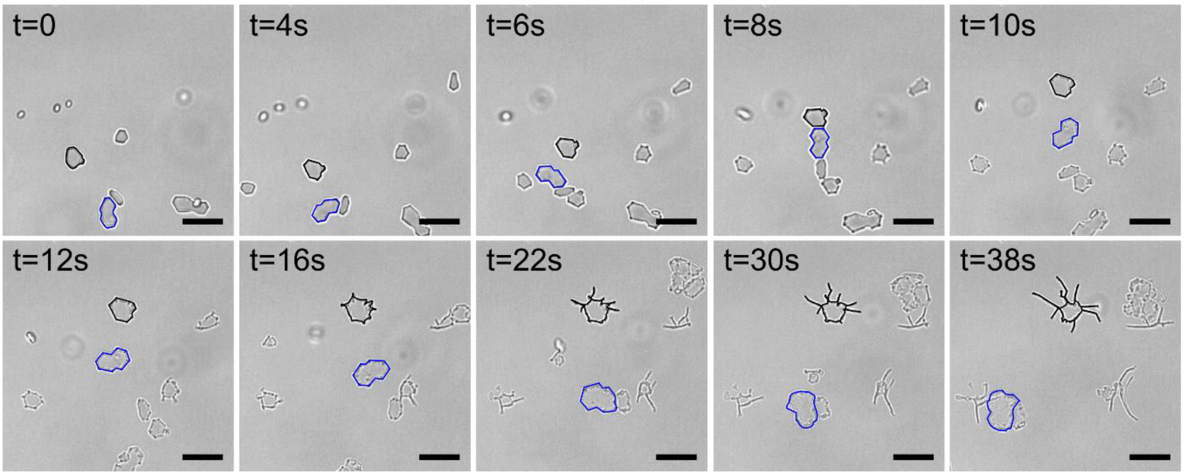

Figure S10. Time-lapse series of optical images of azoTAB:PAA microparticles under blue-light irradiation. *Trans*-azoTAB:PAA particles are initially exposed to blue light in aqueous suspension and then settle with time onto a highly PEGylated glass substrate in the form of hexagonal tablets of *trans/cis*-azoTAB:PAA. Adherence to the surface (at t = 12 s for the particle delineated with the black line) results in the outgrowth of filaments (t = 16-38 s). In contrast, the hexagonal platelet delineated by the blue line retains its shape under blue light during this time period as it remains in suspension and does not therefore adhere to the functionalized glass surface. Scale bars = 10 µm.

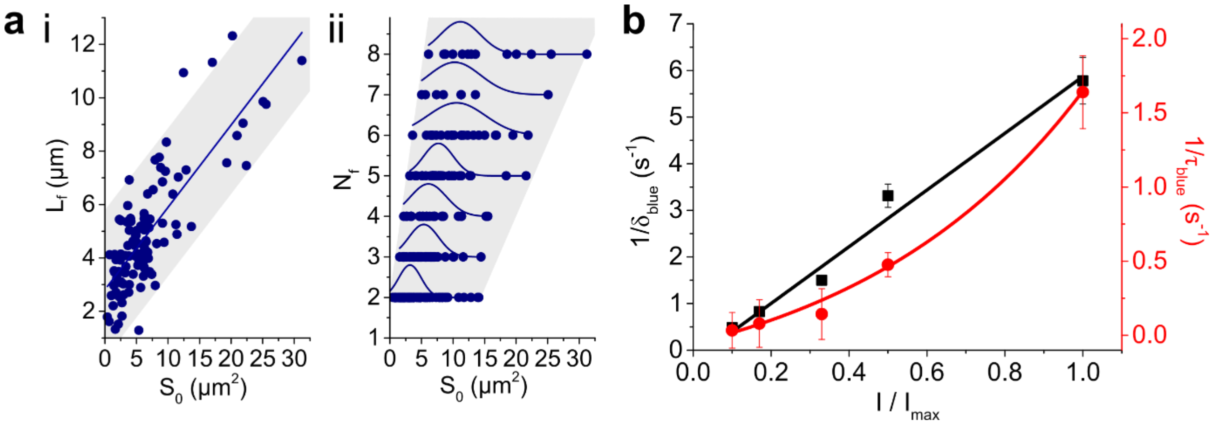

Figure S11. (**a**) Plots showing (**i**) variation of the filament length (L_f) measured at t ~ τ_{blue} as a function of the initial surface area of the all-*trans* microparticles (S_0), and (**ii**) changes in of the number of filaments per particle (N_f) as a function of S_0; data points are fitted with Gaussian distributions. (**b**) Plots showing variation of $1/\delta_{blue}$ (black) and $1/\tau_{blue}$ (red) as a function of light intensity.

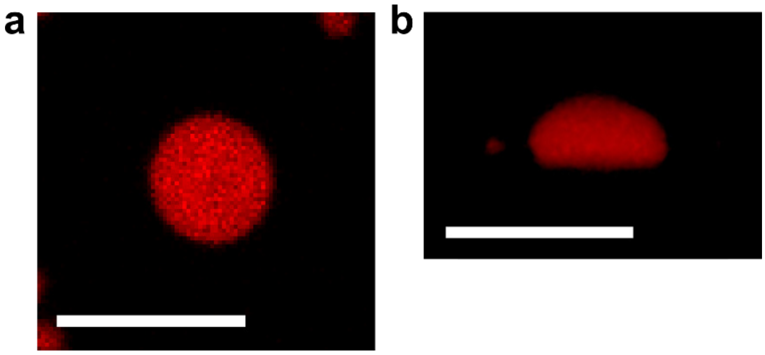

Figure S12. Confocal fluorescence images of UV-adapted *cis*-azoTAB:PAA microparticles containing Nile red and settled on a glass slide showing (a) a circular 2D cross-section and (b) a hemispherical shape when viewed side-on; scale bars = 10 μm.

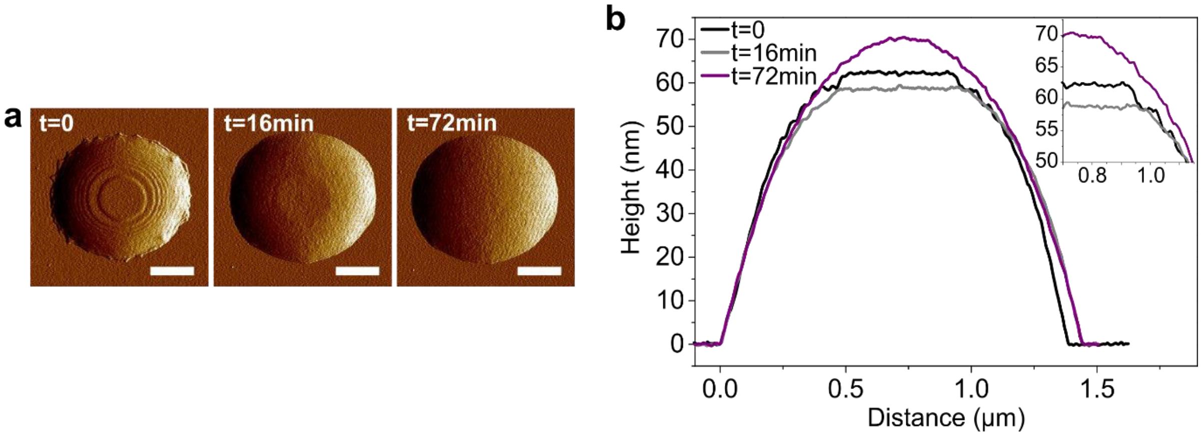

Figure S13. (**a**) Peak force error AFM images of the same azoTAB:PAA microparticle before (t=0; all *trans*) and after 16 or 72 min (all *cis*) *in situ* exposure to UV irradiation; scale bars = 0.5 μm. (**b**) Corresponding height profiles across the particle.

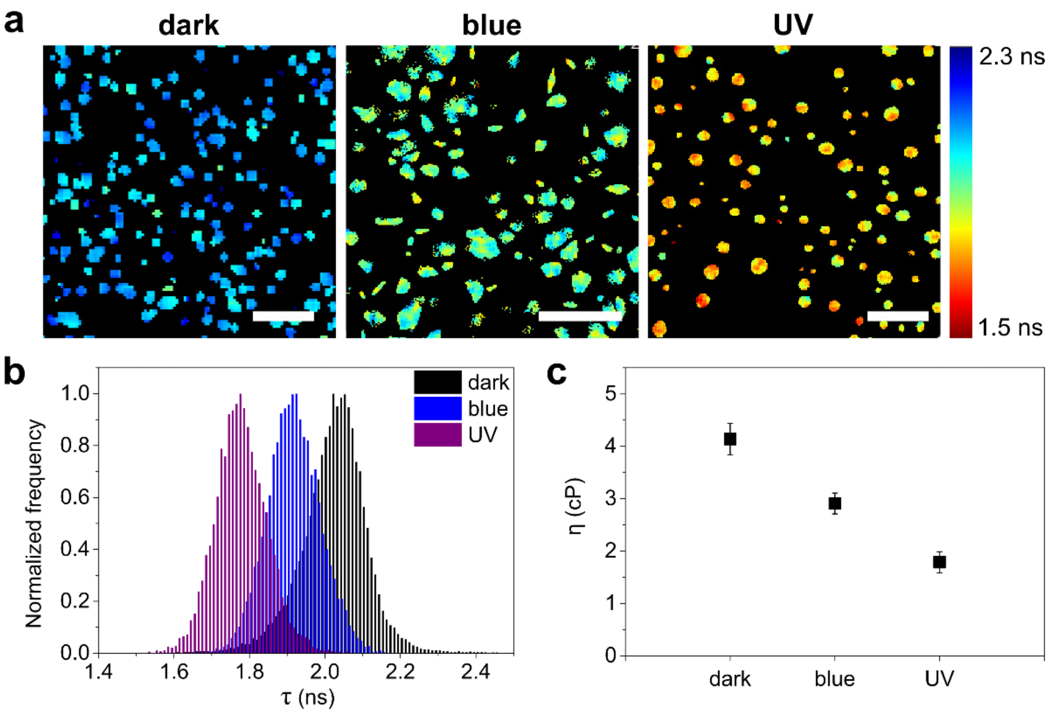

Figure S14. (**a**) Fluorescence lifetime maps for sulforhodamine B-doped hydrated azoTAB:PAA microparticles mounted on a highly PEGylated glass substrate in the dark (left**,** all *trans*), or after blue light (middle**,** *trans/cis*) or UV (right**,** all *cis*) irradiation; colours map different fluorescence lifetimes; scale bars = 10 µm. (**b**) Corresponding fluorescence lifetime distribution histograms extracted from fitting of the FLIM images. (**c**) Plot of viscosities derived from FLIM data for various samples.

Note: azobenzene groups can be involved in fluorescence quenching, so that the values of microviscosity deduced from the fluorescence lifetimes may not reflect the absolute values of the local viscosity. However, the relative differences of microviscosity agree well with the nanomechanical measurements performed on individual particles by AFM.

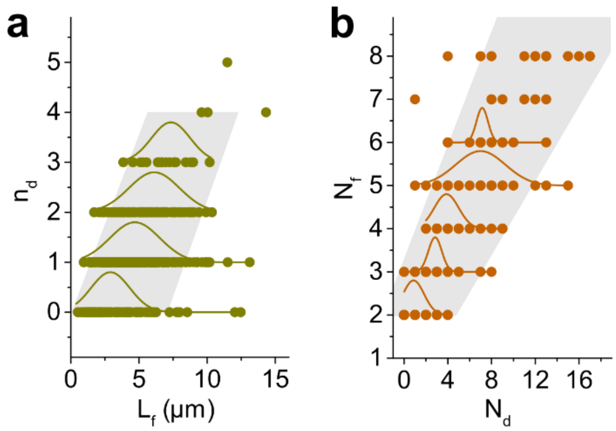

Figure S15. (**a**) Number of *cis*-azoTAB:PAA particles formed per filament (n_d) after UV-induced division as a function of the filament length (L_f); data points are fitted with Gaussian distributions. (**b**) Total number of *cis*-azoTAB:PAA particles formed per initial *trans*-azoTAB:PAA particle (N_d) as a function of the number of filaments (N_f) present in the intermediate multipodal architectures; data points are fitted with Gaussian distributions.

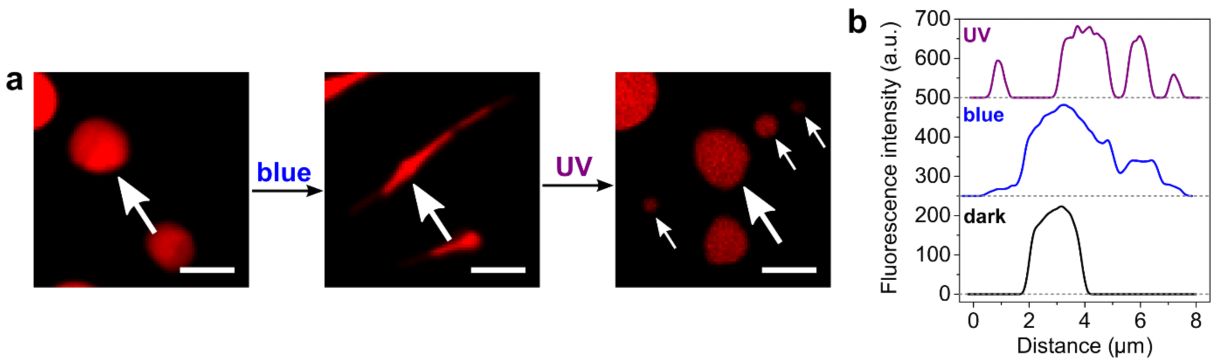

Figure S16. (**a**) Confocal fluorescence images of azoTAB:PAA microparticles containing Nile red during light-induced shape transformations associated with exposure to blue light followed by UV irradiation. The sequestered dye molecule is retained within the surfactant-polyelectrolyte soft solid as it transforms from the irregular *trans*-azoTAB:PAA microparticle (left) to filamentous multipodal *trans/cis*-azoTAB:PAA intermediate (middle) to multiple hemispherical particles of isotropic *cis*-azoTAB:PAA (right); scale bars = 5μm. (**b**) Corresponding intensity profiles across the particle before transformation (dark), after formation of filaments (blue) and after division (UV).
